# Increase in mammography detected breast cancer over time at a community based regional cancer center: a longitudinal cohort study 1990–2005

**DOI:** 10.1186/1471-2407-8-131

**Published:** 2008-05-10

**Authors:** Judith A Malmgren, Mary K Atwood, Henry G Kaplan

**Affiliations:** 1 HealthSTAT Consulting Inc., Seattle, WA, USA; 2 School of Public Health and Community Medicine, Department of Epidemiology, University of Washington, Seattle, WA, USA; 3 Swedish Cancer Institute at Swedish Medical Center, Seattle, WA, USA

## Abstract

**Background:**

Coincident with the advent of mammography screening, breast carcinoma in situ has increased in the US population.

**Methods:**

We conducted a prospective cohort study of all women presenting with primary breast cancer, aged 21–94, and biopsy confirmed Stage 0-IV from 1990–2005 identified and tracked by our registry. Clinical presentation characteristics including age, race, TNM stage, family and pregnancy history, histologic type and method of detection by patient (PtD), physician (PhysD) or mammography (MgD) were chart abstracted at time of diagnosis. Cases with unknown or other method of detection (n = 84), or unusual cell types (n = 26) were removed (n = 6074).

**Results:**

From 1990 to 1998 the percentage of PtD and MgD cases was roughly equivalent. In 1999 the percentage of MgD cases increased to 56% and PtD dropped to 37%, a significant 20% differential, constant to 2005 (Pearson chi square = 120.99, p < .001). Overall, percent TNM stage 0 (breast carcinoma in situ) cases increased after 1990, percent stage I and III cases declined, and stage II and IV cases remained constant (Pearson chi square = 218.36, p < .001). Increase in MgD over time differed by age group with an 8.5% increase among women age 40–49 and 12% increase among women age 50–95. Women age 21–39 rarely had MgD BC. In forward stepwise logistic regression modeling, significant predictors of MgD BC by order of entry were TNM stage, age at diagnosis, diagnosis year, and race (chi square = 1867.56, p < .001).

**Conclusion:**

In our cohort the relative proportion of mammography detected breast cancer increased over time with a higher increase among women age 50+ and an increase of breast carcinoma in situ exclusively among MgD cases. The increase among women currently targeted by mammography screening programs (age ≥ 50) combined with an increase of breast carcinoma in situ most often detected by mammography screening indicates a possible incidence shift to lower stage breast cancer as a result of mammographic detection.

## Background

Breast cancer is the most prevalent and incident type of cancer among women in the United States [1]. In 2003, breast cancer composed 41% of all prevalent cancer cases. Mammography techniques for identification of breast cancer were first developed in the 1960s, the results of mammography screening programs were first published in the 1970s, and recommended screening guidelines were developed in the 1980s [2-5]. Screening guidelines have undergone changes over the past ten years and continue to change almost annually depending on interpretation of published literature. The decrease in mortality risk among screen detected breast cancers has been found to be attributable to a shift to earlier stage and a more favorable prognosis [6-8].

In spite of strong scientific evidence supporting the value of mammography screening, a budget cut to the National Breast Cancer and Cervical Cancer Early Detection Program was proposed in 2006 [9]. In 2007 the American College of Physicians issued recommendations advising women age 40–49 to obtain a clinical assessment of risk before undergoing mammography screening [10]. Recommendations for mammography screening among women aged 70 and older are being reconsidered and the value of mammography screening in older women is being questioned [11,12]. Self breast exam and clinical breast exam have both been questioned as viable screening methods with changing recommendations for their use as screening tools over the past 15 years [13-15].

Our study aims to identify changes and trends in mammography detected breast cancer over time by reviewing primary breast cancer presentation characteristics at a population based comprehensive community cancer care center in a major urban area which is part of the Seattle-Puget Sound Surveillance Epidemiology and End Results (SEER) cancer registry program of the National Cancer Institute [16].

## Methods

We conducted a prospective cohort study of all women presenting with primary breast cancer biopsy confirmed Stage 0-IV from 1990–2005, identified and tracked by our registry. The registry contains all newly diagnosed cases treated at our comprehensive community cancer care center which includes surgical, oncology and radiation therapy clinics. The registry contains detailed information on patient characteristics, method of diagnosis, and stage at diagnosis. Clinical presentation characteristics including age, race, TNM stage, family and pregnancy history, histologic type and method of detection by patient (PtD), physician (PhysD) or mammography (MgD) were chart abstracted at time of diagnosis. Our study contained 6074 cases with unknown or other method of detection (n = 84) and unusual cell types (n = 26) removed.

All data collection was conducted using IRB approved methods and all registry data is stored in a password protected HIPAA compliant database. All analyses were conducted using de-identified data as per IRB and HIPAA guidelines. This project was reviewed and approved by the Institutional Review Board at our community based regional cancer center.

Initial breast cancer (BC) detection method information was obtained by careful review of patient medical records by a certified cancer registrar. The three detection methods were mammography (MgD), physician exam (PhysD) or patient detection (PtD). A mammography detected breast cancer refers to disease discovered by routine mammography in the absence of complaints or known physical findings. The mammogram could have been done as part of a screening program or as a repeat mammogram to verify a previous equivocal mammography finding (diagnostic). Physician detection is defined as initiation of work up for breast cancer by findings discovered by the physician at routine visit or visit for other problems. Patient detected was assigned if the patient detected breast symptoms such as a palpable lump, pain, swelling or bleeding which prompted her to schedule a doctor visit. Patients with self detected tumors may have subsequently had a mammogram or ultrasound done but would still be designated as patient detected BC. The detection method designation was only made when it was certain from the record. AJCC Cancer Staging Manual 6^th ^edition categories and definitions were used for tumor, nodes and metastatic (TNM) cancer staging for all years [17].

SPSS version 14 was used for all statistical analysis [18]. Pearson chi square tests were used for bivariate analysis of dichotomous variables and analysis of variance was used for mean comparisons. All p values are two tailed. Test for trend was run using the binary logistic regression model to produce a p value of the effect of year on detection method [19]. Forward stepwise regression was used for multivariate modeling with mammography detection compared to manually detected BC by patient or physician, as the outcome of interest.

Our institution has been a contributor to the Seattle-Puget Sound SEER registry since 1974–1975. SEER*Stat was used to review SEER modified AJCC 3^rd ^edition (1988+) stage at diagnosis results for the 13 county Seattle-Puget Sound SEER-9 data by year for comparison to our institutions data [20]. A frequency matrix was run using Seattle (Puget Sound) registry and 'breast' site code by year of diagnosis 1990–2002 (n = 42,857) from the SEER limited use file, April 2007.

## Results

From 1990 to 2005, 6074 breast cancer (BC) cases were diagnosed and treated at our institution. Mean age at diagnosis was 57 years ranging from 21 to 94 years. Mean age at diagnosis increased from 56.48 years in 1990–1998 to mean age of 57.68 years in 1999–2005 (F statistic = 12.40, p < .001). Percent Asian, African-American, Hispanic and other non-white race increased from 8.2% in the 1990s to 13.5% in the years 1999 to 2005 (Pearson chi square test = 69.00, p < .001) (table 1).

**Table 1:** Patient/tumor characteristics by mammography detection (n = 6074)

Variable	MgD = no*	MgD = yes	Chi square	p value
	N (%)	N (%)		

	(n = 2884)	(n = 3190)		

**Age**	Row %	Row %		
21–39	427 (88.4%)	56 (11.6%)	558.99	p < .001
40–49	868 (60.0%)	578 (40.0%)		
50–59	668 (40.1%)	996 (59.9%)		
60–69	449 (36.3%)	787 (63.7%)		
70–94	472 (37.9%)	773 (62.1%)		
**Race**				
White	2448 (46.1%)	2866 (53.9%)	35.33	p < .001
Asian	232 (56.9%)	176 (43.1%)		
Black	94 (59.1%)	65 (40.9%)		
Other	60 (61.9%)	37 (38.1%)		
**Diagnosis Year**				
1990	120 (60.9%)	77 (39.1 %)	98.22	p < .001
1991	137 (54.8%)	113 (45.2%)		
1992	132 (53.4%)	115 (46.6%)		
1993	154 (55.8%)	122 (44.2%)		
1994	162 (54.0%)	138 (46.0%)		
1995	157 (52.7%)	141 (47.3%)		
1996	168 (52.7%)	151 (47.3%)		
1997	209 (54.0%)	178 (46.0%)		
1998	230 (53.4%)	201 (46.6%)		
1999	193 (44.0%)	246 (56.0%)		
2000	191 (42.4%)	260 (57.6%)		
2001	166 (39.0%)	260 (61.0%)		
2002	190 (41.3%)	270 (58.7%)		
2003	210 (42.2%)	288 (57.8%)		
2004	250 (42.6%)	337 (57.4%)		
2005	215 (42.3%)	293 (57.7%)		
**TNM stage**				
0	88 (12.1%)	641 (87.9%)	1269.80	p < .001
I	871 (33.1%)	1758 (66.9%)		
II	1161 (64.5%)	640 (35.5%)		
III	638 (82.5%)	135 (17.5%)		
IV	126 (88.7%)	16 (11.3%)		

*combined patient and physician detected breast cancer cases

**Figure 1:**
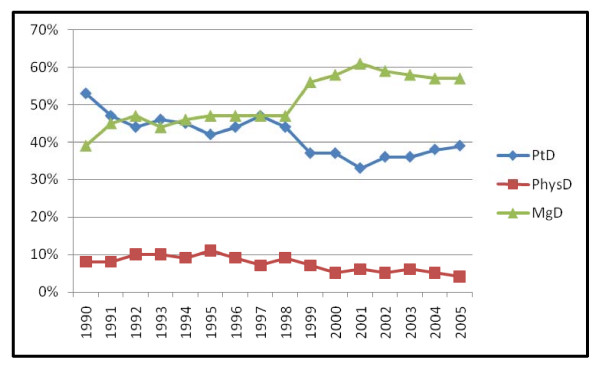
Change in detection method by diagnosis year (n = 6074).

**Figure 2:**
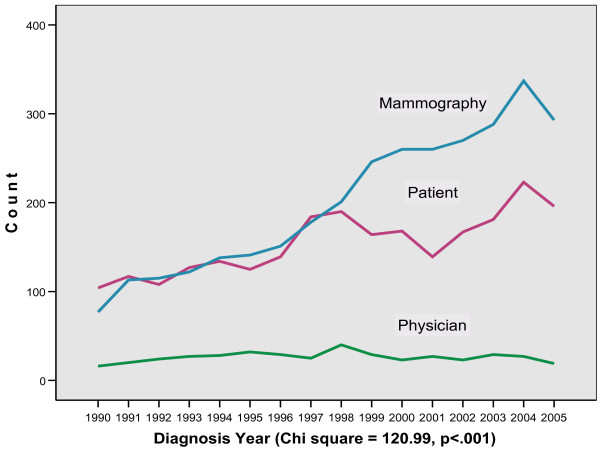
Number of cases by detection method by diagnosis year (n = 6074).

From 1990 to 1998 the percentage of PtD and MgD were roughly equivalent, 45% PtD and 46% MgD. In 1999 the percentage of MgD cases increased to 56% and PtD dropped to 37%, a significant 20% differential that remained constant from 1999 to 2005 (Pearson chi square = 120.99, p < .001) (figure 1 and table 1). The absolute number of PtD cases increased after 1996 and remained relatively constant from 1997 to 2005 with an average of 179 PtD cases per year (figure 2). A test for trend using the p value of the effect of year from the binary logistic regression model of the detection method (mammography detected yes/no) on year was significant, p < .001. The relative percentage of physician detected cases declined from 9.8% in 1993 to 3.7% in 2005 but the absolute number remained constant at approximately 26 cases per year (figure 2).

**Figure 3:**
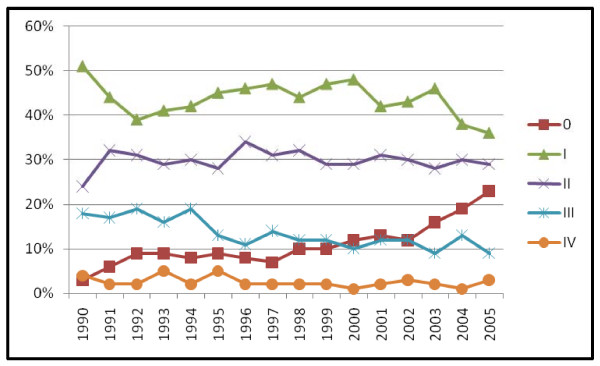
All detection method breast cancer: TNM stage by diagnosis year (n = 6074).

**Figure 4:**
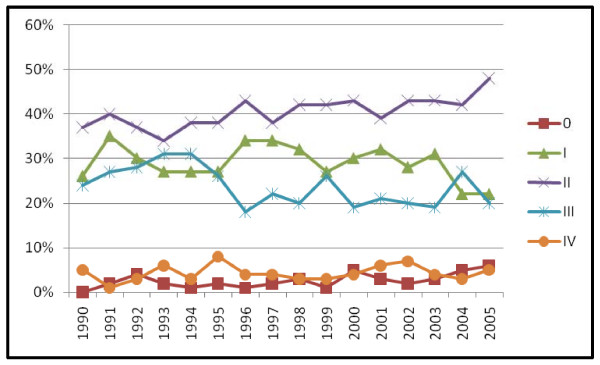
Patient detected breast cancer: TNM stage by diagnosis year (n = 2466).

**Figure 5:**
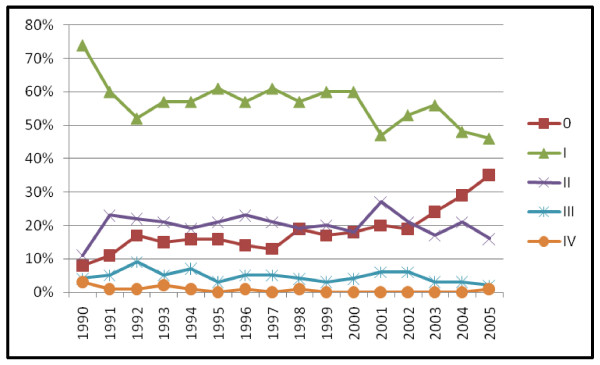
Mammography detected breast cancer: TNM stage by diagnosis year (n = 3190).

Including all detection methods, the relative percentage of TNM stage 0 cases increased after 1990, the percentage of stage I and III cases declined and the percentage of stage II and IV cases remained constant (Pearson chi square = 218.36, p < .001) (figure 3). TNM stage 0 cases in our cohort were all ductal or lobular carcinoma in situ (DCIS, DCIS/LCIS, or LCIS). Histologic type differed significantly by detection method with BC in situ primarily detected by mammography, vs. patient or physician detected cases (table 2). Breast carcinoma in situ increased from 7.9% of all cases in 1990–1998 to 15.3% in 1999–2005 with a steady increase in rate annually as shown in figure 3. The majority of women with TNM stage 0 BC were treated with lumpectomy and radiation (85.6%) with 14.4% receiving mastectomies. When MgD and PtD BC were analyzed separately the increase in TNM stage 0 BC and decrease in stage I BC over time was found to be primarily in the MgD group (MgD chi square test for trend = 152.41, p < .001) (figures 4 and 5).

**Table 2:** Histologic type by mammography detection (n = 6074)

	MgD = no*	MgD = yes	Chi square	p value
	N (%)	N (%)		

	(n = 2884)	(n = 3190)		

	row %	row %		
Carcinoma NOS	37 (84%)	7 (16%)	518.12	.000
Colloid/Mucinous	43 (43%)	58 (57%)		
DCIS	86 (12%)	610 (88%)		
DCIS/LCIS	0	10 (100%)		
LCIS	3 (18%)	14 (82%)		
[BC in situ, all types]	[89 (12%)]	[634 (88%)]		
Ductal	2222 (52%)	2035 (48%)		
Lobular	297 (55%)	242 (45%		
Lobular/Ductal	120 (52%)	109 (48%)		
Papillary	10 (40%)	15 (60%)		
Medullary	27 (90%)	3 (10%)		
Metaplastic	18 (72%)	7 (28%)		
Tubular	21 (21%)	80 (79%)		

*combined patient and physician detected breast cancer cases

**Figure 6:**
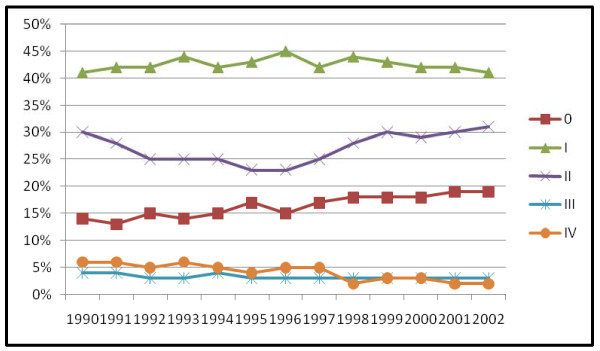
SEER-9 Seattle-Puget Sound region modified AJCC-3 TNM stage by diagnosis year (n = 42,857).

Half as many of the MgD in situ BC cases had mastectomies (13%) compared to 26% of the patient and physician detected tumors (Pearson chi square = 11.44, p < .001). Of 5843 women with tumor size recorded, 68% of the MgD tumors were less than or equal to 1.5 cm and 69% of the PtD and PhysD tumors were larger than 1.5 cm (Pearson chi square = 800.89, p < .001). The MgD in situ BC tumors were significantly smaller, mean tumor size equal to 1.89 cm, compared to 2.49 cm for the PhysD in situ BC and 3.17 cm for the PtD in situ BC tumors (F statistic = 11.17, p < .001). Only 1.8% of the MgD cases were age 21–39 (n = 56) so age 21–39 year olds were not analyzed for tumor size change over time. By diagnosis year, change in tumor size over time for women age 40–49 (18%, n = 546) and age 50+ (80%, n = 2437) were the following: 1) age 40–49, no significant change in tumor size over time (F statistic .857, p=.613), and 2) age 50–94 significant change in tumor size over time with an increase in mean size from 1.22 cm in 1990 to 1.63 cm in 2005 (F statistic = 3.53, p < .001). The largest increase in percentage of MgD cases over time was among women aged 50–95 (12%) followed by an increase of 8.5% among women aged 40–49, with no increase in MgD BC among women 21–39 comparing time periods 1990–1998 and 1999–2005. Increase in mammography detected breast cancer did not differ by race over time. Seattle-Puget Sound SEER-9 registry data for 1990–2002 by TNM stage had a similar increase in TNM stage 0 BC relative to other TNM stage BC increasing from 12% of the total in 1990 to 20% in 2002 (n = 42,857) (figure 6).

In forward stepwise regression analysis with mammography detection as the outcome of interest, order of entry into the model by significance to the outcome was 1) TNM stage, 2) patient age, 3) diagnosis year, and 4) patient race. Women diagnosed between 1999 and 2005 were 1.48 times more likely to have breast cancer found by mammography (95% CI = 1.32, 1.67, p value < .001) in a model corrected for TNM stage, patient age and race. Factors associated with mammography detected BC were 1) stage 0 disease (BC in situ), 2) 50+ years of age, 3) later diagnosis years, 1999 to 2005 compared to 1990–1998, and 4) white race designation (table 1).

## Discussion

In our community based cohort of breast cancer registry cases we observed a steady rate of mammography detected breast cancer from 1990 to 1998 that increased significantly from 1999 to 2005. A significant increase in TNM Stage 0 (in situ) breast cancer was coincident with the increase in MgD BC from a low of 3% in 1990 to a high of 23% in 2005, averaging 8% from 1990 to 1998 and increasing to an average of 15% from 1999 to 2005. The shift to more cases of breast carcinoma in situ over time was exclusively in the mammography detected BC group. Mammography detected breast cancer was found more often in women of white race and was most often less than 1.5 cm in size. Year of diagnosis was a significant predictor of mammography detection indicating an increasing trend towards mammography detection after 1998. We also observed a steady increase in absolute number of breast cancer cases presenting to our institution with the number of physician and patient detected cases remaining constant but an increasing number of mammography detected cases.

Our study is composed of women presenting with a breast cancer diagnosis and detection method identified as patient, mammography or physician. From available medical records we were not able to distinguish if mammography detected cases had participated in regular scheduled mammography screening. In the patient detected breast cancer group we were not able to distinguish between detection by formally trained self breast exam or by incidental findings and we do not know if or when they had prior mammograms. Therefore the patient detected breast cancer group may contain interval cancers in women who were in between mammography screenings but also could contain women who do not have regular mammography screening done.

Nationally there has been a significant increase in the number of women ≥ 40 years of age reporting 'having had a mammogram in the past 2 years' between 1991 and 2001, while more recent results from the Behavioral Risk Factor Surveillance System (BRFSS) identify a slight decline of 2% from 2000 (76.4%) to 2005 (74.6%) [21,22]. The report of a sustained increase in the number of women receiving screening mammograms coincides with our observation of a steady increase in the percentage and number of mammography detected breast cancers.

The observed increase of in situ breast cancer over time among MgD BC cases is remarkable and significant. Although our cohort is relatively small, our observed trends mirror those of the larger geographic area of which we are a subset indicating generalizability of our results. An increase in DCIS over time in the United States has been reported from SEER data [23-25]. As detection method information is not available from SEER data this is the first report to link an observed increase in breast carcinoma in situ directly to mammography detection, confirming previous hypotheses [26]. A stage shift in which more breast cancer would be detected at an earlier stage i.e. a smaller size with participation in mammography screening programs was predicted by Tabar et al and has been observed in the Swedish Two-County study of mammography screening programs [27]. Recently a decline in the rate of invasive breast cancer incidence was reported from Surveillance, Epidemiology and End Results (SEER) cancer registry data for 2003 among women aged >50 years [28]. This observed decline has been attributed to the contemporaneous decline in hormone replacement therapy use [29]. Alternatively the observed decline in invasive breast cancer could be at least partially attributable to an increase in mammography detected breast cancer with more cases of BC in situ and fewer cases of invasive breast cancer.

In our cohort the majority (>80%) of advanced stage BC were in the patient or physician detected groups. Advanced breast cancer (TNM stage III and IV) was fairly constant over time in our cohort which may be due to 1) patient detected breast cancer in young women not eligible for screening, 2) patient detected breast cancer in women who may not participate in a screening program, 3) patient detected interval breast cancer of an aggressive nature in women who may participate in a mammography screening program, and 4) BC not detectable by mammography. Factors associated with patient detected breast cancer are addressed in a previous paper by the same authors [15].

## Conclusion

In our cohort we observed an increase in the relative proportion of mammography detected breast cancer over time with a higher relative increase among women age 50+ and an increase of breast carcinoma in situ exclusively among mammography detected cases. Relative to mammography detection, the number of patient detected breast cancers also increased over time to a lesser degree and the number of physician detected breast cancers remained constant. The increasing representation of women aged >50 years who are currently targeted by mammography screening programs combined with an increase of in situ BC which is more likely to be detected by mammography screening indicate a partial shift to a less advanced form of breast cancer. The change over time to more mammography detected and lower stage breast cancer may indicate movement towards the achievement of mammography screening goals. These results support retention of screening programs and recommendations as well as increased awareness of the potential importance of other detection methods.

## Abbreviations

BC: breast cancer; DCIS: ductal carcinoma in situ; MgD: mammography detected; PtD: patient detected; PhysD: physician detected; SEER: Surveillance, epidemiology and end results; TNM: tumor, nodes and metastasis.

## Competing interests

The authors declare that they have no competing interests.

## Authors' contributions

All three authors made 1) substantial contributions to the conception and design, acquisition of data, or analysis and interpretation of the data, 2) have been involved in drafting the manuscript or revising it critically, and 3) have given final approval of the version submitted for publication. Each author has participated sufficiently to take public responsibility for appropriate portions of the content.

## Pre-publication history

The pre-publication history for this paper can be accessed here:


